# The Material Growth and Characteristics of Transition Metal Oxide Thin Films Based on Hot Wire Oxidation Sublimation Deposition Technology

**DOI:** 10.3390/ma18174083

**Published:** 2025-08-31

**Authors:** Fengchao Li, Qingguo Kang, Zhenwei Kang, Tengteng Li, Jiangang Yu, Haibing Qiu, Ting Liang, Cheng Lei

**Affiliations:** 1 School of Semiconductor and Physics, North University of China, Taiyuan 030051, China; 2 State Key Laboratory of Widegap Semiconductor Optoelectronic Materials and Technologies, North University of China, Taiyuan 030051, China; 3 State Key Laboratory of Extreme Environment Optoelectronic Dynamic Measurement Technology and Instrument, North University of China, Taiyuan 030051, China

**Keywords:** transition metal oxides, hot wire oxidation sublimation deposition, material growth, optoelectronic characterization, hole transport layers

## Abstract

Transition-metal oxides (TMOs) possess pronounced optoelectronic properties and are widely exploited in photovoltaics and photocatalysis. Here, we introduce a hot wire oxidation sublimation deposition (HWOSD) that directly converts elemental Mo and W into amorphous MoO_x_ and WO_x_ films on various substrates. Scanning electron microscopy and atomic force microscopy reveal uniform thickness and conformal coverage over textured and planar surfaces. X-ray photoelectron spectroscopy indicates high oxygen contents with stoichiometric ratios of 2.94 (MoO_x_) and 2.91 (WO_x_). Optical measurements show transmittances > 94% across 400–1200 nm, yielding optical band gaps of 1.86 eV (MoO_x_) and 2.67 eV (WO_x_). The conductivities of MoO_x_ and WO_x_ were 2.58 × 10^−6^ S cm^−1^ and 5.14 × 10^−7^ S cm^−1^ at room temperature, and the TMO/Si surface potential differences are 200 mV and 114 mV, respectively. Minority-carrier-lifetime measurements indicate that MoO_x_ films confer an additional passivation benefit to the i a-Si:H/c-Si/i a-Si:H stack. Annealing of MoO_x_ and WO_x_ realized their phase transition from an amorphous state to a polycrystalline state, with changes in their optical transmittance in the visible light region. Investigation of the photovoltaic performances of MoO_x_ and WO_x_ as HTLs deposited by HWOSD demonstrates their excellent electronic functionality in optoelectronics. These results establish HWOSD as a scalable, low-temperature method to fabricate high-quality TMO films and expand their potential in advanced optoelectronic devices.

## 1. Introduction

Transition metal oxides (TMOs) possess tunable energy bands, excellent light absorption capabilities, high carrier mobility, and wide spectral absorption, which have great potential in photovoltaics, photocatalysis, and electronic devices [1,2,3]. The conduction-band bottom of the TMO is sufficiently negative and its valence-band top appropriately positive, jointly satisfying the requirements for hydrogen and oxygen evolution [4,5]. Moreover, TMOs exhibit exceptional chemical robustness, rendering them highly suitable for sustained photocatalytic water splitting [6]. The high specific capacity and rapid charge transport enhance the energy density and cycle life of supercapacitors [7,8,9]. TMOs can also achieve rapid and reversible coloration under an electric field [10,11,12], improving the durability of electrochromic devices. In photovoltaics, TMO thin films, characterized by a near-optimal band gap, high optical transmittance, and outstanding thermal stability, serve as an ideal selective transport layer in solar cells [13,14]. Consequently, achieving high-quality TMOs has become a key technology for further advancing photovoltaics, photocatalysis, and electronic devices.

Within the realm of TMOs-enabled applications, molybdenum oxide (MoO_x_) and tungsten oxide (WO_x_) have garnered substantial research interest due to their unique physicochemical properties. MoO_x_ is an indirect bandgap with a bandgap of 2.9–3.2 eV [15]. Its work function reaches 6.8 eV, increasing with the increase of the oxygen/molybdenum ratio [16]. WO_x_ is also an indirect bandgap semiconductor with a relatively wide bandgap (3.2 eV) and is n-type, exhibiting high chemical stability and high conductivity [17,18]. And it can also be achieved by combining amorphous tungsten oxide with crystalline tungsten oxide to form a WO_3_ film. The high cycle stability of crystalline WO_3_ and the high ion storage and transmission capacity of amorphous WO_3_ can be utilized to enhance the overall performance and work function of the film [19,20].

The properties of TMO thin films in electronic materials are largely influenced by the deposition methods, including magnetron sputtering [21], thermal evaporation [22], and the sol-gel method [23], hot wire oxidation-sublimation [24,25,26,27], etc. In an O_2_/Ar plasma, a WO_3_ film was deposited by 60 W RF magnetron sputtering of a W target. XRD showed that it was in the hexagonal phase (100) and (200). WO_3_ films with different morphologies can be fabricated by using magnetron sputtering technology combined with oblique angle deposition technology. According to the UPS analysis, the deviation of the base position led to a decrease in the work function, and this resulted in an increase in the minimum value of the conduction band that approaches the reduction potential [27]. MoO_x_ films were deposited by RF reactive magnetron sputtering at 4 mTorr while varying O_2_ flow, whose XPS confirmed increased stoichiometry with higher oxygen content, raising the work function to 5.92 eV, the highest yet reported for reactively sputtered MoO_x_ [28]. Although magnetron sputtering routinely delivers dense, high-quality TMO films, the accompanying plasma bombardment frequently inflicts subtle substrate damage, elevating interfacial defect densities and, consequently, compromising ultimate device performance.

A 200 nm WO_3_ film was deposited at room temperature by thermal evaporation and subsequently annealed at 200, 400, and 500 °C for 1 h. X-ray diffraction confirmed the emergence of a highly oriented orthorhombic phase with preferential (001) orientation, UV–vis and photoluminescence spectroscopy revealed a progressive narrowing of the optical band gap from 3.25 eV to 2.60 eV [29]. The thermal evaporation has enabled the growth of uniform MoO_x_ films on quartz and glass substrates, which were found to have amorphous characteristics by XRD analysis [30]. Thermal evaporation mitigates plasma-induced substrate damage, yet the inevitable pellet agglomeration of transition-metal-oxide powders tends to reduce both growth rate and source utilization, while subtly lowering the TMO work function.

Using hexachlorotungsten as the precursor, WO_3_ films were grown by the sol-gel method. Their structure, composition, and optical properties were characterized by SEM, XRD, and XPS. Cyclic voltammetry, chronoamperometry, and in-situ transmission measurements revealed that post-annealing the WO_3_ film attained an electrochemical reversibility of 0.81, substantially surpassing its as-deposited counterpart [31]. MoO_x_ films were prepared by the sol-gel method using MoO_3_ powder and NH_3_ or H_2_O_2_ solution as precursors at room temperature. Then, they were directly treated with ultraviolet ozone without the need for high-temperature annealing. Through AFM, XPS, and UPS measurements, it was confirmed that the films were uniform, dense, had a stable chemical structure, and high work function [32]. While solution-based routes excel in process simplicity and cost-effectiveness, residuals from the precursors can increase interfacial trap densities and subtly compromise the TMO work function.

The hot-wire oxidation–sublimation deposition (HWOSD) demonstrates exceptional promise for fabricating transition-metal oxides. High-quality WO_x_ films were fabricated via HWOSD by precisely regulating oxygen-vacancy density at 850 °C tungsten filament and 4–16 sccm O_2_, with XPS/UPS revealing a direct correlation between vacancy concentration and work function, enabling tunable values from 4.97 to 5.41 eV [24]. With increasing the oxygen flow ratio, the ratio of W^6+^ increases while W^5+^ decreases, concomitantly elevating the work function of WO_x_ [25]. Prior investigations had already demonstrated the viability of the hot-wire oxidation–sublimation deposition (HWOSD) technique for synthesizing molybdenum oxide (MoO_x_) thin films [26]. Nevertheless, a systematic and comparative study that unifies the synthesis–structure–property relationships of HWOSD-grown WO_x_ and MoO_x_ remains absent, motivating the integrated investigation.

This work introduces a comprehensive and comparative account of HWOSD-enabled synthesis and in-depth characterization of WO_x_ and MoO_x_ thin films, whose morphology, electronic properties, and thermal stability were characterized by SEM, AFM, XPS, and XRD. The silicon heterojunction solar cells using MoO_x_ or WO_x_ as Hole selective transport layer were fabricated and showed excellent performance, offering robust theoretical guidance for TMO thin films deposition and its device application.

## 2. Experimental Details

### 2.1. Hot Wire Oxidation Sublimation Deposition

**Figure 1:**
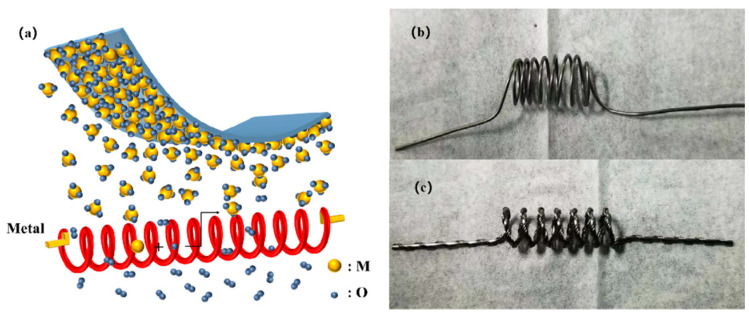
(**a**) Schematic diagram of hot-wire oxidation sublimation deposition method, (**b**) Molybdenum wire (99.9%, diameter d = 1.0 mm), (**c**) Tungsten wire (99.7%, Two tungsten wires with diameter d = 1.0 mm are intertwined).

Hot wire oxidation sublimation deposition (HWOSD) was used to fabricate MoO_x_, and WO_x_, thin films, as shown in Figure 1: (a) In a vacuum chamber, oxygen and high temperature (in range of 600–1100 °C) hot wires of transition metals (such as: Mo and W) were reacted, generating their oxides on surfaces, which sublimated simultaneously and spread into the whole chamber in the form of molecular clusters, then deposited on the substrate surface, forming the transition metal oxide thin film.

The HWOSD system was constructed by retrofitting a conventional hot-wire chemical vapour deposition (HWCVD) chamber. A 1.0 mm-diameter molybdenum (ZhongNuo Advanced Materials Co., Ltd., Beijing, China, 99.95%) wire or a 1.0 mm-diameter tungsten (ZhongNuo Advanced Materials Co., Ltd., Beijing, China, 99.99%) wire—length 120 mm, mounted between two copper electrodes—was positioned 10 cm beneath the substrate holder (Figure 1). TMO films were grown according to the following sequence: (1) The chamber was evacuated to 4 × 10^−5^ Pa to ensure a contamination-free environment. (2) The DC current was ramped at 40 mA/min (Mo) or 5 A/min (W) until the filament reached the target temperature, monitored in situ by an infrared pyrometer (±5 °C accuracy). (3) High-purity O_2_ (99.999%) was introduced via a mass-flow controller; the desired working pressure was established and maintained by adjusting the throttle valve. (4) The substrate holder was rotated at 100 rpm, and the shutter opened to initiate deposition. The film thickness was controlled to ±1 nm by timing the shutter interval after calibrating the growth rate with stylus profilometry. (5) After deposition, the filament current was switched off, residual O_2_ was pumped out, and the samples were retrieved for further characterization or device processing.

Such a process of fabricating thin film materials by the HWOSD method contained the following stages: (1) Oxidation reaction: where a wire generated high temperature under the electric current due to its own electrical resistance, providing heat for its oxidation reaction with oxygen. The high-temperature transition metal on the surface of the hot wire was oxidized to generate a transition metal oxide. The current was controlled for the oxidation rates and the film quality. (2) Physical sublimation: high temperature h influenced the sublimation rate, which in turn affected the growth rate of the oxide material on the substrate. (3) Diffusion deposition: The transition metal oxide sublimated from the surface of the hot wire and entered into the vacuum chamber, with random diffusion for the deposition of the transition metal oxide film on the substrate surface.

In this work, the HWOSD technique enables conformal deposition of transition-metal-oxide (TMO) thin films on a variety of substrates while maintaining identical geometric boundary conditions for reproducible growth. Standard soda-lime glass slides (25 mm × 25 mm × 3 mm, Schott D263T, SCHOTT AG, Mainz, Rhineland-Palatinate, Germany) were employed for optical-transmittance measurements and annealing studies of TMO films. The polished crystalline-Si wafers (p-type, 1–10 Ω·cm, 300 µm) were used to assess film-thickness uniformity (stylus profilometry), crystal structure (XRD), oxidation state (XPS), contact potential difference (SKPM), and minority-carrier lifetime. Alkaline-textured c-Si wafers (1–3 µm pyramid height) served to demonstrate the conformal (coverage-preserving) capability of HWOSD and to fabricate a solar cell. All substrates underwent identical pre-treatment: glass slides were ultrasonically cleaned in acetone, isopropanol, and 18.2 MΩ cm de-ionized water (10 min each) and dried with N_2_; Both polished and textured silicon substrates were cleaned via the standard RCA sequence and subsequently dried with N_2_. Each substrate was mounted on a rotating holder positioned 10 cm beneath the hot wire at 100 rpm to ensure uniform TMO film thickness across the entire substrate area.

### 2.2. Characterization

To systematically evaluate the performance of the as-deposited MoO_x_ and WO_x_ thin films, the following comprehensive characterization techniques were employed:

Film thicknesses of MoO_x_ and WO_x_ were quantified with an ERUKER-Dektak XT stylus profiler (vertical resolution 0.1 nm, Bruker Corporation, Billerica, MA, USA). Optical properties were derived from UV–Vis transmittance spectra recorded on a Hitachi UV-4100 spectrophotometer (Hitachi High-Tech Science Corporation, Tokyo, Japan), whose spectrophotometer across 175–3300 nm with 0.2 nm UV/Vis and 1 nm NIR wavelength accuracy, using a 60-mm integrating sphere to ensure sub-0.01% baseline noise and reliable film data down to 200 nm. In this work, all spectra were acquired within the 300–1800 nm window relevant to solar-energy harvesting and device transparency. Surface morphology was imaged with a Hitachi SU-8010 field-emission scanning electron microscope (Hitachi High-Technologies Corporation, Tokyo, Japan) (FE-SEM, resolution 0.8 nm at 10 kV). The surface structure of the film material was analyzed using the NTEGRA Spectra II atomic force microscope (AFM, NT-MDT Spectrum Instruments, Moscow, Russia, Z-noise ≤ 0.1 nm, RMS in 10–1000 Hz bandwidth, XYZ closed-loop sample scanner 100 μm × 100 μm × 10 μm). The surface contact potential between the film and the Si substrate was evaluated by using the NTEGRA Maximus Kelvin Probe Microscopy (KPM, NT-MDT Spectrum Instruments, Moscow, Russia) with a lateral resolution of ≤50 nm and potential sensitivity of ≤1 mV over a ±10 V range, operating in single-pass mode at ambient conditions. Grazing-incidence X-ray diffraction (GIXRD) was performed on 50-nm MoO_x_ and WO_x_ films using a Rigaku SmartLab SE diffractometer (Rigaku Corporation, Tokyo, Japan, Cu Kα, λ = 1.5406 Å) with a fixed incident angle of 0.5° and a 2θ scan range of 10–80°, achieving a resolution of 0.01° and a depth sensitivity of 5–100 nm. Electronic structure information for identical 50-nm films on polished Si substrates was obtained by X-ray photoelectron spectroscopy (XPS, Thermo Fisher Scientific, Waltham, MA, USA, monochromatic Al Kα, 1486.6 eV, energy resolution ≤ 0.48 eV). The passivation quality of the various film architectures was evaluated by measuring the minority-carrier lifetime of corresponding Si wafers via quasi-steady-state photoconductance (QSSPC) using a Sinton WCT-120 system (Sinton Instruments, Boulder, CO, USA, 10 ns-10 ms calibrated range, 40–230 mm wafer). Finally, in-plane electrical conductivity was determined by depositing coplanar Ag electrodes onto the films on glass and performing current–voltage.

### 2.3. Fabrication and Characterization of Solar Cells

An n-type (100) silicon wafer, 200 µm thick with a resistivity of 1–5 Ω·cm, served as the starting substrate. After alkaline texturing to create a light-trapping surface, the wafer was subjected to the standard RCA cleaning sequence. A 6-nm-thick hydrogenated amorphous silicon (a-Si:H) passivation layer was then deposited on both faces at 200 °C via PECVD. Subsequently, an indium tin oxide (ITO) film 70–100 nm thick was sputtered onto the illuminated side using magnetron sputtering, followed by the evaporation of Ag front electrodes through a metal shadow mask. On the rear side, Ag electrodes were formed by thermal evaporation as the back contact. The photoelectric performance of solar cells is typically evaluated using the light and dark J-V curve under standard conditions (AM1.5, 100 mW/cm^2^, 25 °C).

## 3. Results and Discussion

### 3.1. Morphology of MoO_x_ and WO_x_ Thin Films

The microscopic morphology of TMO thin films determines the quality of the interface formed with other materials to a great extent. MoO_x_ and WO_x_ films were deposited on polished silicon wafers and random pyramid substrates, respectively, to observe the morphological characteristics of oxide films on different substrates.

**Figure 2:**
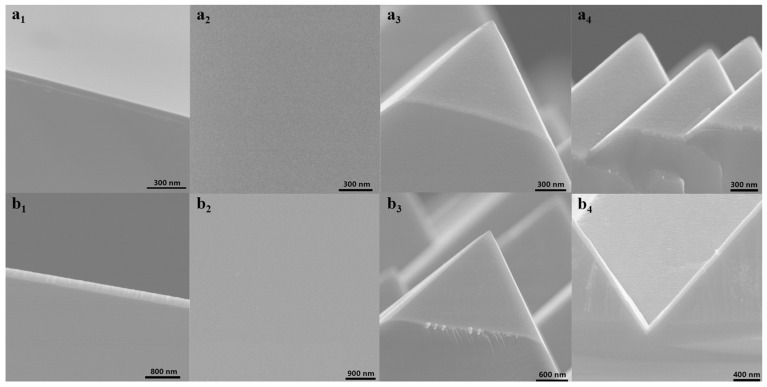
SEM images of molybdenum oxide (**a_1_**–**a_4_**), tungsten oxide (**b_1_**–**b_4_**) on different substrates: 1 & 2, cross-sectional and top-view on a polished silicon wafer; 3 & 4 on textured silicon wafers.

Figure 2(a_1_–a_4_) are SEM images of MoO_x_ thin film fabricated on different substrates under optimized deposition conditions (hot wire temperature of 1050 °C and oxygen pressure of 0.2 Pa) by HWOSD. For deposition of MoO_x_ on a polished silicon wafer, Figure 2(a_1_) displays a cross-sectional view of the MoO_x_ film with uniformed thickness. As can be seen from Figure 2(a_2_), the top view of the MoO_x_ film deposited on the polished silicon wafer exhibited uniform distribution on the surface of the polished silicon wafer. The MoO_x_ films on textured silicon wafers can form a good coverage of the random pyramid light trapping structure, as shown in Figure 2(a_3_,a_4_).

WO_x_ (under W wire temperature of 1200 °C and oxygen pressure of 1.0 Pa) were fabricated on different substrates under the optimized preparation process. Figure 2(b_1_) proved that HWOSD can also achieve uniform growth of tungsten oxide thin films on polished silicon wafers. On the surface of the silicon wafer with random pyramid texture, WO_x_ can also achieve the “conformal” deposition of the light trapping structure, thereby creating the necessary conditions for the subsequent formation of an ideal device interface.

**Figure 3:**
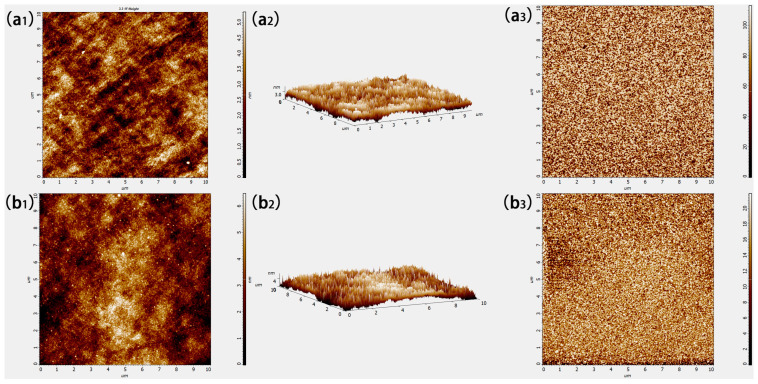
AFM height (**a_1_**,**b_1_**), 3D height (**a_2_**,**b_2_**) and phase (**a_3_**,**b_3_**) images of MoOx (**a_1_**–**a_3_**) and WOx (**b_1_**–**b_3_**) films.

The atomic force microscopy (AFM) was used to characterize the roughness and phase of each film under optimal processing conditions, as shown in Figure 3.

Figure 3(a_1_,a_2_) are the 2D and 3D height graphs of the MoO_x_ films deposited on the surface of the polished silicon wafer, respectively. The root mean square roughness (RMS, Sq) in the scanning range of the MoO_x_ film was 1.10 ± 0.02 nm, and the average roughness was 0.88 nm. The phase diagram of MoO_x_ is shown in Figure 3(a_3_), and the scale on the right is the phase difference between the reflected laser signal of the needle tip and the excitation signal of the needle tip caused by the sample, which represents the sensitivity of different elements in the sample to the tip. The light and dark distributions in the phase diagram in this study respectively represent the distribution of Mo and O elements in the sample, which are evenly distributed in the MoO_x_ film fabricated by HWOSD.

The height diagram of WO_x_ film on the surface of the polished silicon wafer is shown in Figure 3(b_1_,b_2_). The RMS roughness Sq of the WO_x_ films was 1.32 ± 0.02 nm, and the average roughness was 1.06 nm. The phase diagram of WOx film (b_3_) also showed that the WO_x_ film prepared in this study had a uniform phase in Figure 3(b_3_).

By analyzing the morphological characteristics of transition metal oxides via SEM and AFM, MoO_x_ and WO_x_ thin films deposited by HWOSD possessed uniform thickness and a smooth surface. They could be achieved by “conformal” deposition on the textured surface, which was important to the subsequent application of MoO_x_ and WO_x_ in solar cells.

### 3.2. Structural Characteristics of TMO Thin Films

**Figure 4:**
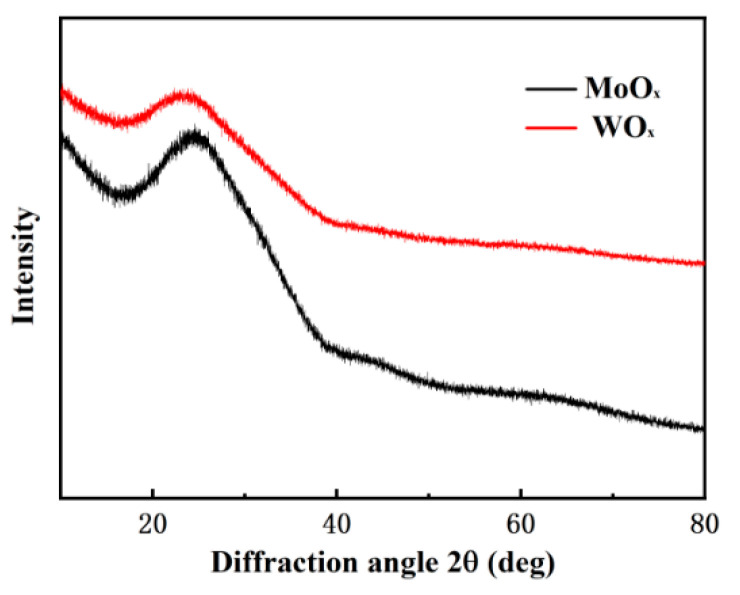
X-ray diffractogram of transition metal oxides (MoO_x_ and WO_x_).

The structural characteristics of MoO_x_ and WO_x_ were characterized by X-ray diffraction (XRD), are shown in Figure 4. The X-ray diffractogram of MoO_x_ and WO_x_ films on the glass substrate which be seen that the transition oxide films appeared similar, and no characteristic peaks were presented, indicating an amorphous film, which was related to the relatively low substrate temperature (<200 °C). It is tentatively inferred that the broad, weak humps centred at 2θ ≈ 24.4° (MoO_x_) and 23.8° (WO_x_) correspond to the short-range order of Mo-O and W-O polyhedra, respectively, and are in quantitative agreement with the nanometre-scale correlation lengths calculated from the Scherrer equation (~0.8 nm), further supporting the amorphous nature of the films.

### 3.3. The Electronic Structure of TMO Thin Films

**Figure 5:**
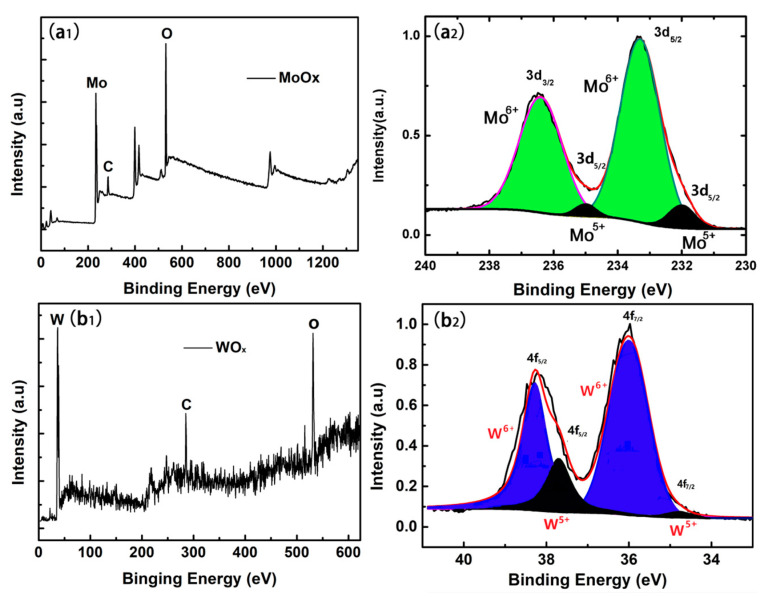
X-ray photoelectron spectroscopy (XPS) of MoO_x_ (**a_1_**) and WO_x_ (**b_1_**), and spectra for: Mo 3d core level in MoO_x_ films (**a_2_**), W 4f core level for WO_x_ films (**b_2_**).

X-ray photoelectron spectroscopy (XPS) was used to analyze the electronic structure characteristics of oxide films. Figure 5(a_1_,b_1_) presents the XPS spectra of MoO_x_ and WO_x_ within the entire scanning range, respectively. According to the XPS standards, the approximate position of the element contained in the oxide is found, providing a specific energy interval for the second accurate scan.

The XPS spectrum of the 3d electron of the Mo element in the range of binding energy (BE) of 230 to 240 eV, the molybdenum oxide sample was subjected to a second fine sweep, and the XPS peaking software was used for the obtained data (XPSPEAK-41) can be processed to obtain Figure 5(a_2_). The positions of the BE at 233.3 and 236.4 eV correspond to the 3d_5/2_ and 3d_3/2_ orbital electrons of Mo^6+^, respectively, and the characteristic peaks at 232 eV and 235 eV originate from the 3d orbital electrons of Mo^5+^. By fitting and normalizing the characteristic peaks, the content ratio between Mo^6+^ and Mo^5+^ (shaded area ratio) in MoO_x_ can be obtained, and the stoichiometric number x in MoO_x_ is estimated to be 2.94 [26]. This value is higher than the result of 2.79–2.86 in MoO_x_ reported in the previous literature, which shows that the higher oxidation state of molybdenum element in MoO_x_ fabricated by HWOSD, implying that the thin film material could have a larger work function.

In the same way, the second fine sweep of the characteristic peak of the W element in WO_x_ thin film material can be obtained as shown in Figure 5(b_2_). The BE at the positions of 36.0 eV and 38.2 eV corresponds to +6 valence of W ions occupying 4f_7/2_ and 4f_5/2_ electron orbitals, respectively. At the positions where the BE are 34.8 eV and 37.0 eV, the +5 valent W ions occupying 4f_7/2_ and 4f_5/2_ electron orbitals are presented. Similarly, fitting and normalizing the characteristic peaks resulted in the ratio of 5.33:1 between the W element of +6 and +5 valence in tungsten oxide prepared by this HWOSD. It is inferred that the value of x in WO_x_ is roughly 2.91, which also shows a higher degree of oxidation of tungsten element in the tungsten oxide prepared by the HWOSD method.

The HWOSD-derived MoO_2.94_ and WO_2.91_ exhibit elevated M^6+^/M^5+^ ratios, evidencing a more highly oxidised, defect-poor surface that favours an increased work function for hole-selective contacts.

### 3.4. Optical Properties of MoO_x_ and WO_x_

**Figure 6:**
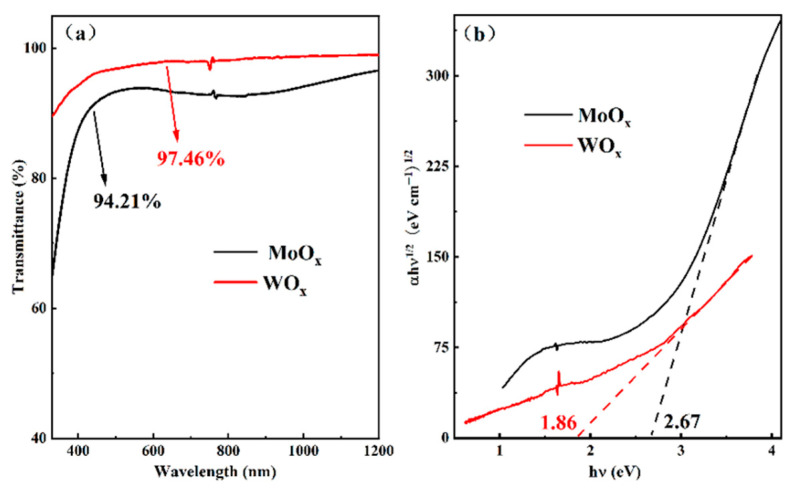
The transmittance (**a**) and optical band gap (**b**) of TMOs.

Figure 6a is a comparison diagram of the transmittance of films of MoO_x_ and WO_x_ with the same thickness (15 nm) fabricated under optimal conditions. It can be seen that TMOs with the same thickness have different light transmittance. The average transmittance of WO_x_ film in the wavelength range of 300–1200 nm is as high as 97.46%, indicating that it has excellent optical performance. Compared to WO_x_ thin films, MoO_x_ films have a bit worse light transmittance with an average transmittance of 94.21%. In general, these two TMOs all have high optical transparency. Their absorption coefficient and optical band gap of each material were analyzed. The absorption coefficient of thin film material α can be expressed as:

(1) $$\alpha =\frac{1}{d}ln(\frac{1-R}{T})$$

Where T is the transmittance of the film, d is the thickness of the film, and R is the reflectance of the film material.

The relationship between the light absorption coefficient of the material and the photon energy *hν* follows the Tauc law near the absorption edge:

(2) $$\alpha h\nu =\beta {\left(h\nu -Eg\right)}^{r}$$

where β is a constant; r is an optional parameter, the indirect band gap material is 2, and the direct one is 1/2; Eg is the optical band gap of the material; *hν* is the photon energy.

Assuming that the film reflectivity R is 0, and the MoO_x_ and WO_x_ films are amorphous silicon materials, referring to the studies of M. Anwar [33], SH Mohamed [34,35], and S. Sindhu [36], and Kumari et al. [37], and knowing the indirect band gap for the three oxides, our calculate resulted in a Tauc curve, as shown in Figure 6b. Extrapolating the linear portion of the curve to the abscissa, the value corresponding to the intersection point leads to the optical band gap of the material. From Figure 6b, the optical band gaps of MoO_x_ and WO_x_ were 2.67 eV, and 1.86 eV, respectively. M. Anwar and other researchers found that the optical band gap of the MoO_x_ film was affected by the thickness of the film itself [37], the deposition temperature, and the annealing, varying between 2.3 and 2.9, which was similar to that of the MoO_x_ film measured in this work. According to studies by Corsin Battaglia et al. [38,39]. The forbidden band width of the amorphous molybdenum oxide film was 3.0–3.3 eV [40], but the optical band gap of the molybdenum oxide film fabricated in this experiment was 2.61 eV. The random fluctuation of the internal field caused by the disordered structure in the crystalline film, which in turn caused the optical band gap of the amorphous silicon film to change, referring to the studies of Cody, G., D., et al. [41]. It may also be due to the existence of a defect state energy level in the forbidden band of the molybdenum oxide film, which could absorb incident light of a certain wavelength, and the optical band gap was smaller than the forbidden band width [42]. The existence of defect states in the molybdenum oxide film provided a necessary channel for the transport of holes at the interface [43,44]. SH Mohamed and other studies have shown that the optical band gap of WO_x_ films prepared by sputtering increased with the increase of oxygen pressure [35]. When the oxygen pressure increased from 0.01 Pa to 0.52 Pa, the optical band gap of the tungsten oxide prepared also increased from 1.56 eV to 3.18 eV [45]. The fabrication of tungsten oxide in this work had an oxygen pressure of 1.0 Pa, being different from those reported in the literature, which also belonged to a comparable thin film deposition technology under low oxygen pressure. It could be seen from these results that the optical band gap of the oxide was affected by the structural characteristics of the film, the preparation conditions, and the content of elements inside the film.

### 3.5. Electrical Characteristics of MoO_x_ and WO_x_

**Figure 7:**
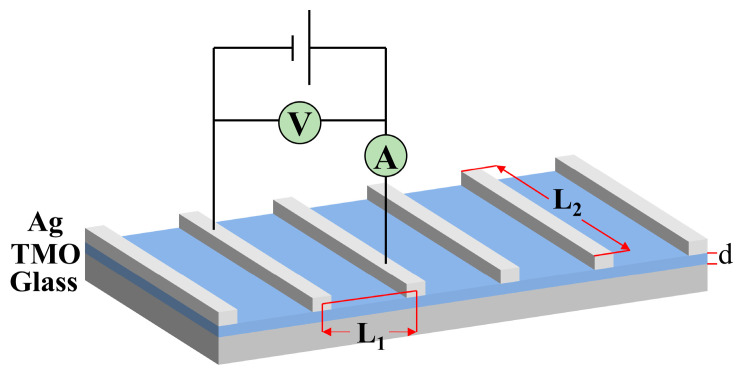
Schematic diagram of transition metal oxide conductivity measurement.

As seen in Figure 7, the HWOSD was used to fabricate a TMO film with a certain thickness on a glass substrate, and a grid electrode was prepared by thermal evaporation on the surface of such films, for testing electrical conductivity. Their conductivity σ (with the unit of S/cm) was obtained by Equation (3), with the corresponding current value when applying the electrical potential between the two electrodes:

(3) $$\sigma =\frac{I*L_{1}}{U*d*L_{2}}$$

where d (cm) is the thickness of the film material; U is the voltage (V); I is the current (A); L_1_ and L_2_ (cm) are the electrode spacing width and electrode length, as show in Figure 7. The conductivity obtained for MoO_x_ and WO_x_ films were 2.58 × 10^−6^ and 5.14 × 10^−7^ S/cm, respectively.

**Figure 8:**
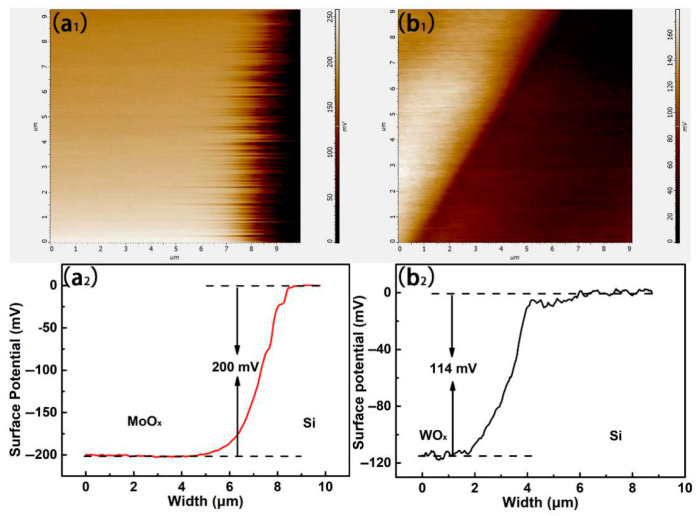
(**a_1_**) Potential distribution of MoOx film on silicon wafer surface. (**a_2_**) The surface potential difference between MoO_x_ and silicon in the z-axis direction. (**b_1_**) The potential distribution of WO_x_ film on silicon wafer surface. (**b_2_**) The surface potential difference between WO_x_ and silicon in the z-axis direction.

The surface potential of MoO_x_ and WO_x_ on polished silicon wafers was measured by Kelvin Probe Microscopy (KPM), and the energy band relationship at the interface between the transition oxides and the silicon wafer was qualitatively analyzed. The measurement results are shown in Figure 8.

The working principle of the Kelvin probe microscope is: when two different samples are in contact, the Fermi level will be in the same position at the contact interface. But due to the different work functions of the materials, the energy bands are bent at the interface, leading to the redistribution of different charges that generate forces with the probes of the device. It was such tested forces that provided us with the surface potential and the work function of the materials.

Different potential distributions between MoO_x_ and n-Si are illustrated in Figure 8(a_1_), according to the brightness and darkness of the colors. Figure 8(a_2_) stands for the potential value on the Z axis obtained from Figure 8(a_1_). It can be seen that the potential on the MoO_x_ side is about 200 mV lower than that on the Si plate side. It showed that higher work function and lower Fermi level of MoO_x_ than those of n-Si resulted in redistribution of surface charges and therefore the bending of their energy bands at interfaces, building up a potential barrier for electron transport, while benefiting the selective hole transport when they moved towards the MoO_x_ side based on the attraction from MoO_x_’s low work functions.

The potential distribution of the WO_x_ film in contact with the Si wafer is shown in Figure 8(b_1_), which follows similar mechanisms to the interactions between MoO_x_ and Si wafers. Figure 8(b_2_) similarly presents the contact potential difference between WO_x_ and Si in the Z-axis direction. Comparing Figure 8(a_2_,b_2_), the contact potential difference of MoO_x_ (200 mV) was found significantly higher than that of WO_x_ (114 mV), which could be inferred that the MoO_x_ film might cause a greater band bending. The different work functions of 6.9 eV for MoO_x_ and 6.5 eV for WO_x_, respectively, could be the right reason for causing the difference in the bending of the crystalline silicon band.

A direct comparison reveals that MoO_x_ delivers a larger contact-potential difference than WO_x_, translating into steeper band bending that enhances hole selectivity while suppressing electron recombination.

**Figure 9:**
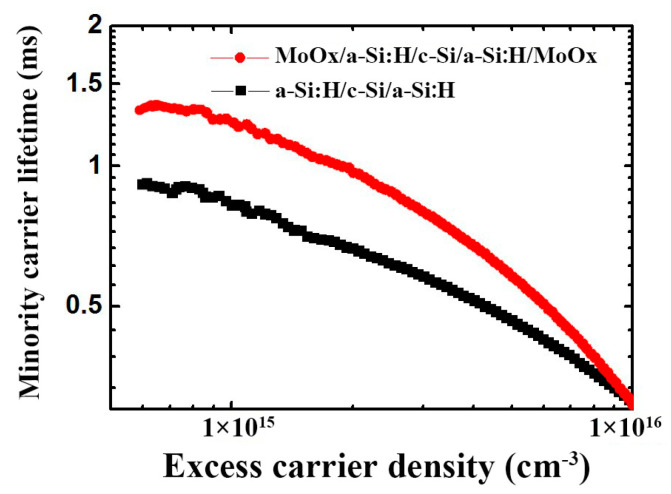
Measured effective lifetimes for samples with the structures of i a-Si:H/c-Si/i a-Si:H and MoO_x_/i a-Si:H/c-Si/i a-Si:H/MoO_x_ as a function of excess carrier density in the range of 7 × 10^14^ cm^−3^ ~ 1 × 10^16^ cm^−3^.

Figure 9 shows the curve of minority carrier’s lifetime with carrier concentration in n-type CZ silicon wafers with i a-Si:H or MoO_x_/i a-Si:H composite layers deposited on both sides [26]. Compared with the passivation of amorphous silicon, the symmetric structure of i: a-Si/MoO_x_ (14 nm) covered on both sides of the silicon wafer causes a longer minority lifetime in their injection concentration range of 7 × 10^14^ cm^−3^~1 × 10^16^ cm^−3^, with 1.3 ms as the highest for MoO_x_/i a-Si:H/c-Si/i a-Si:H/MoO_x_ can reach. This indicated that MoO_x_ had a strong field passivation effect on silicon wafers; that is, a larger work function of the MoO_x_ film could cause a larger band bending at the contact interface with the Si wafer, thereby reducing the recombination of carriers near the contact. Such results could also be corroborated by the test result of the contact potential of the front surface.

Direct lifetime measurements on MoO_x_/Si/MoO_x_ stacks were unattainable, indicating a high density of interfacial defect states. Inserting i a-Si:H between MoO_x_ and Si suppresses these defects and simultaneously exploits the strong field-effect passivation of MoO_x_, yielding a 1.3 ms peak lifetime. Future efforts must therefore focus on defect-state management at the MoO_x_/Si interface to unlock the full potential of TMO-based contact engineering.

### 3.6. Effect of Annealing on Optical and Structural Properties of TMO Thin Films

Considering that the TMO films may suffer the temperature changes when being applied to devices and future possible optimizations on their film properties, the effect of annealing temperature on molybdenum oxide (MoO_x_) and tungsten oxide (WO_x_) films was studied. The two thin film materials deposited on the glass substrate were rapidly annealed at different temperatures using a fast-annealing apparatus. The morphology, transmittance, and structure of the thin films were studied by means of optical microscope, UV-Vis absorption, and XRD methods.

**Figure 10:**
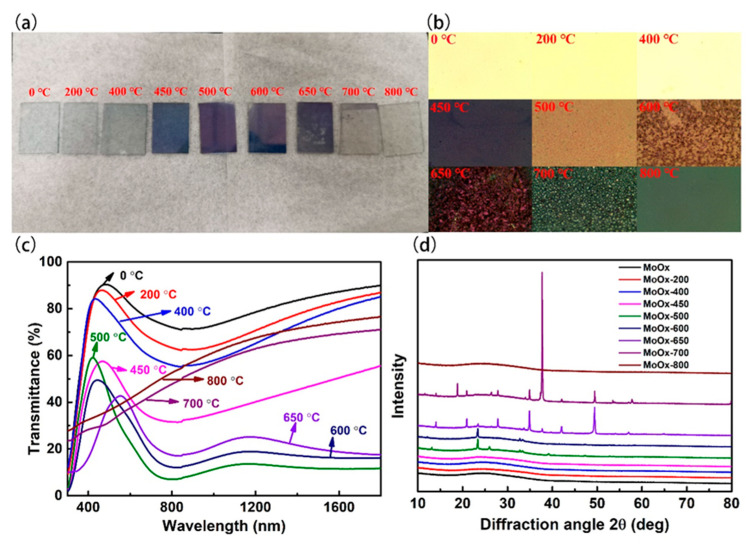
Photos (**a**), optical images (**b**), transmittance (**c**), and XRD diffractograms (**d**) of MoO_x_ films at different rapid-annealing temperatures.

First, under the protection of argon, the molybdenum oxide film was rapidly annealed at 200, 400, 450, 500, 600, 650, 700, and 800 °C, respectively, for 60 s. The morphology, transmittance, and XRD pattern of the films are shown in Figure 10.

As can be seen from Figure 10a,b, when the annealing temperature was less than 400 °C, the appearance of the MoO_x_ film had no obvious changes. As the annealing temperature increased from 450 to 600 °C, the films’ color gradually deepened, and the metallographic microscope showed that the MoO_x_ films gradually appeared granular, implying some structural changes. Further increasing the annealing temperature led to the disappearance of the MoO_x_ film from the substrates. When the annealing temperature reached 800 °C, the granular MoO_x_ had completely disappeared.

Figure 10c shows the transmittance of MoO_x_ at different rapid annealing temperatures. With the annealing temperature increased, the transmittance of the MoO_x_ film as a whole decreased firstly and then increased. When the annealing temperature was raised from 200 to 500 °C, the transmittance of the film dropped to a minimum; a possible reason was that the structure change of the molybdenum oxide film affected its transmittance to some extent. When the rapid-annealing temperature continued to rise, the film’s transmission rate rose continuously because the higher annealing temperature might cause the decomposition or sublimation of MoO_x_ film, exposing the glass substrate, thereby increasing the transmission rate.

From the XRD pattern of Figure 10d, it can be seen that when the annealing temperature was <450 °C, the structure of the MoO_x_ film remained unchanged, still being amorphous. When the rapid-annealing temperature rose to 500 °C, the XRD pattern of MoO_x_ presented diffraction peaks at 13.04°, 23.38°, 25.94°, 39.13°, 47.35°, 52.77°, 39.13°, 47.23°, 52.76°, respectively, corresponding to crystal planes of (0 0 1), (1 0 0), (0 0 2), (0 0 3), (2 0 0), (1 0 −4), and (2 1 0) for MoO_3_ monoclinic phase. When the annealing temperature was 600 °C, the disappearance of characteristic diffraction peaks at 13.04°, 25.94°, 39.13°revealed the vanishing of (0 0 1), (0 0 2), and (0 0 3) crystal planes. When increasing the annealing temperature to 650 °C, the characteristic peak at 23.38° was found much less pronounced, and the one characteristic peak at 32.65° disappeared, meanwhile generation of new diffraction peaks at 13.88°, 20.88°, 27.88° suggested newly formed (6 4 0), (10 4 0), and (6 6 1) crystal planes of MoO_2.8_. And the ones at 34.89°, 37.69° proved the presence of (1 −2 7) (3 −2 4) crystal planes of Mo_9_O_26_, while the one at 57.01° stood for the (14 1 1) crystal plane of Mo_4_O_11_. Continued to increase the temperature to 700 °C, the crystal plane of (3 −2 4) for Mo_9_O_26_ claimed the preferred crystal orientation, corresponding to a peak at 37.66°, accompanied by a peak at 18.74° exhibiting (−4 0 1) crystal plane of Mo_4_O_11_. In summary, as the annealing temperature rose, the amorphous MoO_x_ film generated monoclinic MoO_3_ crystals at 500 and 600 °C. As the temperature continued to rise to 650 and 700 °C, MoO_2.8_, Mo_9_O_26_, and Mo_4_O_11_ appeared; it could be seen that the oxygen element in the MoO_x_ film had a tendency to detach from the film. Literature showed that the reduction of X in MoO_x_ might cause a decrease in the work function of the MoO_x_ film. When it continues to increase to 800 °C, the MoO_x_ thin film in the glass has completely decomposed and escaped the glass substrate, as shown in Figure 10a.

As reported in the literature, an appropriate amount of oxygen was in need to be to maintain a higher work function of the MoO_x_ film by thermal evaporation technique using MoO_x_ powders as the source, which could be attributed to the desorption of oxygen element from MoO_x_ at high temperature [46].

**Figure 11:**
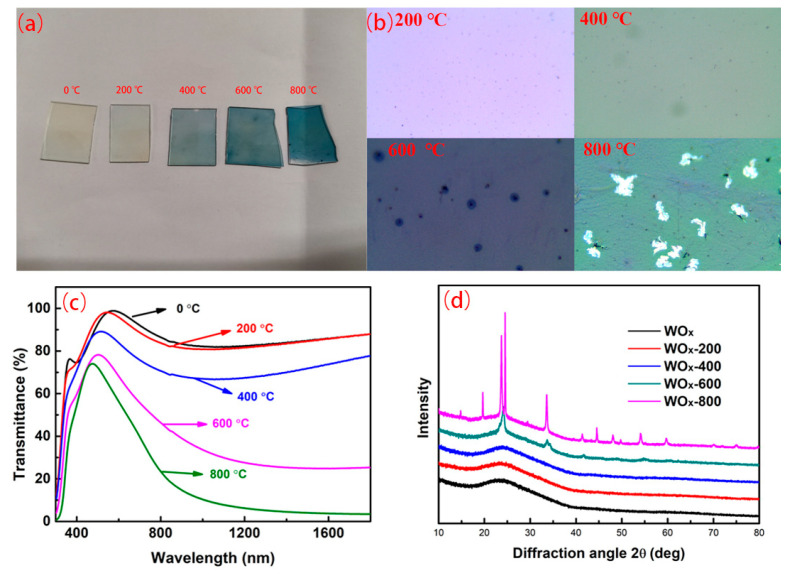
Photos (**a**), morphology under optical microscope (**b**), transmittance (**c**), and XRD pattern (**d**) of WO_x_ films at different rapid annealing temperatures.

Using the same method and process, the rapid annealing performance of WO_x_ film was studied, as shown in Figure 11. It was seen from Figure 11a that rapid annealing gradually changed the color of the WO_x_ film from light to dark. Figure 11b confirmed agglomeration phenomenon at some positions of the WO_x_ film as the annealing temperature gradually rose up to 600 °C. When the annealing temperature was further increased to 800 °C, the WO_x_ film had partially peeled off. The position of the film was seen as a “wrinkle”, suggesting a stress change inside the film.

Figure 11c shows the effect of different annealing temperatures on the transmittance of the WO_x_ films. As the rapid-annealing temperature increased, the transmittance of the WO_x_ film gradually decreased, implying that it might cause structural changes in the WO_x_ film. As can be seen from Figure 11d, when the temperature lay between 200 and 400 °C, the WO_x_ film still maintained an amorphous state. When the rapid- annealing temperature was rose to 600 °C, peaks of diffraction at 23.96°, 34.08°, 42.11°, 49.04°, 55.25°, and 61.07°, respectively, corresponding to (1 0 0), (1 1 0), (1 1 1), (2 1 0), (2 1 0), and (2 1 1) crystal planes. Continued to raise the annealing temperature to 800 °C, the characteristic peaks at 23.72°, 33.60°, 41.32°, 44.68°, 48.03°, 54.15°, 59.75°, and 70.04°, were recognized as crystal planes of (1 1 0), (2 0 0), (2 0 1), (2 1 1), (2 2 0), (3 1 0), (3 1 1), and (4 0 0) for WO_2.9_. When the annealing temperature reached 800 °C, the phenomenon of X reduction in WO_x_ was also found.

In a word, HWOSD-grown MoO_x_ and WO_x_ films exhibit markedly different thermal-stability behaviors. MoO_x_ remains amorphous below 450 °C, crystallises into monoclinic MoO_3_ at 500 °C, and then follows a progressive reduction sequence-MoO_3_-MoO_2.8_-Mo_9_O_26_ -Mo_4_O_11_, until complete sublimation at 800 °C. This trajectory is driven by the intrinsically lower melting/boiling points and higher oxygen volatility of molybdenum oxides. The accompanying optical signature is a transient drop in transmittance during crystallization, followed by a sharp rise as the film disappears. WO_x_, in contrast, retains an amorphous structure up to 400 °C and crystallises into WO_3_ at 600 °C. Continued heating to 800 °C produces only a slight reduction to WO_2.9_, accompanied by densification and partial peel-off rather than catastrophic loss. Consequently, WO_x_ offers a 200–300 °C wider thermal window than MoO_x_. The difference translates directly to interface stability: MoO_x_’s steeper band bending (200 mV contact-potential difference) and higher work function provide superior hole selectivity at low temperature, whereas WO_x_’s 114 mV offset and work function remain stable above 600 °C. Thus, MoO_x_ serves as an optimal low-temperature hole-selective layer, while WO_x_ is the material of choice for devices subjected to prolonged high-temperature processing.

### 3.7. The Performance of Heterojunction Cells with MoO_x_ and WO_x_ as HTLs

**Figure 12:**
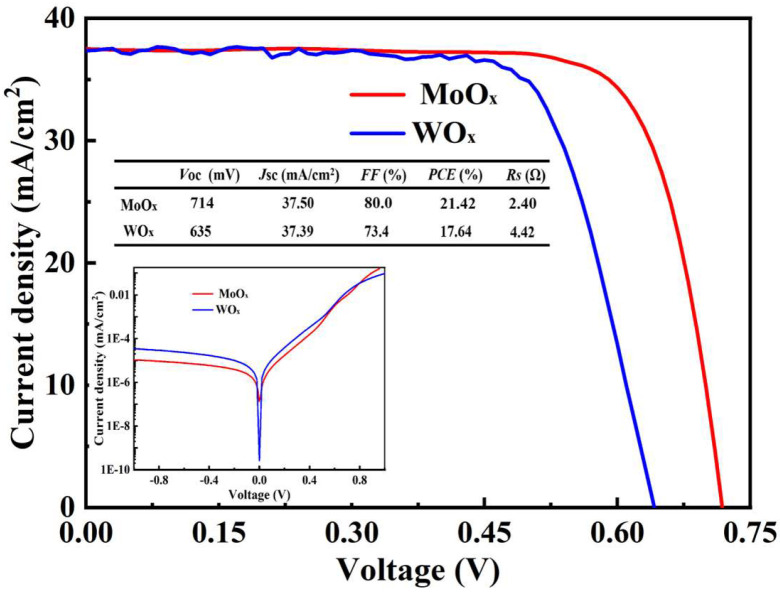
The light and dark *J-V* curves of the heterojunction solar cells with MoO_x_ and WO_x_ as HTLs.

To investigate the performance of transition metal oxide thin films prepared by HWOSD in device applications, heterojunction solar cells were fabricated using MoO_x_ and WO_x_ as hole transport layers (HTLs), and their device performance was evaluated, as depicted in Figure 12.

Based on the observations and experimental results, both types of solar cells exhibit relatively excellent performance. However, the SHJ solar cell employing MoO_x_ as the hole transport layer (HTL) outperforms those utilizing WO_x_ as HTLs. The *V*_OC_ of heterojunction solar cells with WO_x_ as HTLs was lower than that of MoO_x_ solar cells. The factors that affected *V*_OC_ mainly included the work function of TMO and interface characteristics. It is generally believed that the higher the TMO work function, the greater the bending degree of the energy band, which finding is consistent with the results of the Kelvin probe microscope. It can be seen from the reverse saturation current density was MoO_x_ < WO_x_, which could exhibit the density of interface defect states to a certain extent.

The FF of the device is mainly affected by the series resistance. The Rs of MoO_x_ solar cells solar cell was the smallest, which was 2.40 Ω, followed by 4.42 Ω for WO_x_ solar cells, respectively. Considering that the conductivity of the TMO material exerts a substantial impact on the Rs of these devices, the results of the FF and Rs provide compelling validation for the measured electrical conductivity of MoO_x_ and WO_x_ films.

The MoO_x_-equipped cell delivers Voc = 714 mV, FF = 80% and Rs = 2.40 Ω, outperforming the WO_x_ cell (Voc = 635 mV, FF = 73.4%, Rs = 4.42 Ω). The 79 mV Voc gain stems from MoO_x_’s higher work function, which induces steeper band bending and lowers reverse saturation current (J_0_, MoOx < J_0_, WOx). The lower Rs reflects superior TMO conductivity. These results confirm that interface passivation and defect-state control remain the primary levers for further device optimisation.

## 4. Conclusions

In this work, HWOSD enabled the reproducible deposition of MoO_x_ and WO_x_ thin films, and conducted a systematic, side-by-side evaluation of their structural, optical, and electronic properties. Characterization techniques such as SEM, AFM, and XRD revealed that the oxide films exhibit smooth surfaces, uniform thickness, and excellent conformal coverage over textured substrates. XPS analysis indicated that the oxygen-to-metal ratios in MoO_x_ and WO_x_ films were 2.94 and 2.91, respectively. Both oxide films demonstrated good optical transmittance, with optical bandgaps of 2.67 eV for MoO_x_ and 1.86 eV for WO_x_. Electrical property evaluations showed that the films possess favorable conductive characteristics and good contact potential differences with silicon wafers. Heterojunction solar cells employing these oxide films as hole-selective transport layers exhibited promising performance parameters. The results suggest that the TMO thin films prepared by the HWOSD method possess excellent electronic properties and hold great potential for broader applications in the fabrication of photocatalysis, flexible optoelectronics, and roll-to-roll manufacturing. Nevertheless, their long-term thermal stability awaits rigorous validation, while precise doping for oxygen-vacancy control and advanced interface engineering remain pivotal for future optimization.

## Data Availability

The original contributions presented in this study are included in the article. Further inquiries can be directed to the corresponding authors.
